# A 10-year cross-sectional retrospective study on Kawasaki disease in Iranian children: incidence, clinical manifestations, complications, and treatment patterns

**DOI:** 10.1186/s12879-021-06046-2

**Published:** 2021-04-19

**Authors:** Payman Sadeghi, Anahita Izadi, Sayed Yousef Mojtahedi, Leila Khedmat, Mohsen Jafari, Azadeh Afshin, Pourya Yarahmadi, Effat Hosseinali Beigi

**Affiliations:** 1 Department of Pediatrics, Bahrami Hospital, Tehran University of Medical Sciences, Shahid Kiaee Street (Ghasem Abad), Damavand Street, Tehran, 1641744991 Iran; 2 Department of Pediatric Infection Disease, Tehran University of Medical Sciences, Tehran, Iran; 3 Department of Pediatric Nephrology, Bahrami Children Hospital, Tehran University of Medical Sciences, Tehran, Iran; 4 Health Management Research Center, Baqiyatallah University of Medical Sciences, Tehran, Iran; 5 Faculty of Medicine, Tehran University of Medical Sciences, Tehran, Iran; 6 Department of Pediatric Intensive Care Unit, Bahrami Children Hospital, Tehran University of Medical Science, Tehran, Iran

**Keywords:** Kawasaki disease, Children, Coronary artery abnormality, Gastrointestinal complication, Intravenous immunoglobulin, Clinical marker

## Abstract

**Background:**

Kawasaki disease (KD) as an acute, systemic vasculitis is the leading cause of acquired heart disease in children under the age of 5 years.

**Methods:**

A 10-year cross-sectional retrospective study was designed to assess 190 Iranian children with KD during 2008–2018. Demographic data, clinical and laboratory manifestations from the onset of symptoms to diagnosis, clinical signs and symptoms, and subsequent treatments were evaluated to predict hospitalization stay, complications, and response to treatment.

**Results:**

Children with KD had a male-to-female ratio of 1.18:1 and an average age of 36 months. There was an insignificantly more incidence of KD in cold seasons. The most frequent symptoms were fever (92.6%), oral mucus membrane changes (75.8%), bilateral bulbar conjunctival injection (73.7%), polymorphous skin rash (73.2%), peripheral extremity changes (63.7%), and cervical lymphadenopathy (60.0%). The rate of gastrointestinal, cardiac, joint, and hepatic complications was determined to be 38.4, 27.9, 6.8, and 4.2%, respectively. 89.5% of patients received intravenous immunoglobulin (IVIG) plus aspirin as the first line of treatment, while, 16.3% of them needed an extra second line of treatment. Significantly low serum sodium levels and high platelet counts were detected in KD patients with cardiac complications. Cardiac complications often were more encountered in patients who did not respond to the first line of treatment. Higher platelet count, lower serum sodium amount, and C-reactive protein (CRP) level were significantly associated with a need for an additive second line of treatment. A significant relationship between hospitalization stay and hemoglobin level was found.

**Conclusion:**

As most of the clinical manifestations and complications were following other reports released over the past few years, such data can be confidently used to diagnose KD in Iran. Seasonal incidence and a positive history of recent infection in a notable number of patients may provide clues to understand possible etiologies of KD. Laboratory markers can successfully contribute to health practitioners with the clinical judgment of the need for additional treatments, possible complications, and hospitalization duration.

## Introduction

Kawasaki disease (KD), also known as mucocutaneous lymph node syndrome, usually occurs in pediatric populations. More than 80% of patients are younger than 5 y [1]. The Kawasaki disease was the first time in 1967 reported by Dr. Tomisaku Kawasaki among 50 children in Tokyo, Japan [2, 3]. The annual incidence of Kawasaki syndrome among American children is 17.5–20.8 per 100,000 [3, 4]. Besides, the incidence rate of KD in Northeast Asian countries such as Japan, South Korea, and China is 10–30 times more than that in North America and Europe [5]. For instance, the annual incidence of KD in Japanese children is more than 300 per 100,000 [6]. During 1997–2002, the incidence rate of KD among Iranian children was 7.4 per 100,000 [7]. In recent years, epidemiological studies revealed that the occurrence rate of KD has been increasing among Iranian children [8].

This disease with the effect on medium-sized arteries manifests as persistent fever, erythema of the lips and oral mucosa, bilateral non-purulent conjunctivitis, skin rashes, swelling and redness of the hands and feet, and cervical lymphadenopathy and usually follows with a short period of respiratory or gastrointestinal symptoms [1, 4, 5]. As cardiac complications are the most concerning ones, it is essential to adopt efficient methods to diagnose and treat this self-limited disease [9]. If no treatment is performed, the development of coronary artery aneurysms (CAAs) may observe in 15–25% of children with KD [10]. Kawasaki syndrome can also lead to unusual abnormalities such as renal involvement (e.g., pyuria, proteinuria, tubular disturbances, tubulointerstitial nephritis, and renal failure), hydrops of the gallbladder, gastrointestinal ischemia, cranial nerve palsy, jaundice, petechial rash, shock syndrome, febrile convulsions, and encephalopathy or ataxia [11–13].

A high prevalence of CAAs has been recently reported even among KD infants aged younger than six months [14, 15]. Tulloh et al. [16] not only reported a considerable incidence of KD among children in rural areas of the UK and Ireland, but also pointed out that the rate of CAAs was higher compared to other countries despite receiving intravenous immunoglobulin (IVIG) therapy. Furthermore, Jindal et al. [17] showed longer delays in the diagnosis of KD and higher development of CAAs among older Indian children. Therefore, the choice of efficient treatments in children with KD of all ages is necessary to avoid the development of coronary artery abnormalities. The timely CAAs-reducing treatment is high-dose acetylsalicylic acid (ASA) and IVIG [18]. IVIG in the acute stage is typically administered over 10–12 h as a single infusion along with oral ASA. However, the efficacy of high-dose ASA in the acute management of KD was questioned by introducing IVIG as the standard therapy to care KD patients [19]. Besides, some randomized controlled studies showed that the concurrent use of corticosteroids (CSs) and IVIG as an initial treatment for KD could remarkably reduce CAAs without any adverse coronary outcomes [20, 21].

To the best of our knowledge, the incidence and epidemiology of KD and its drug treatment were fewer studied in Iranian children [8, 22–24]. Therefore, this study aimed to evaluate clinical features, main complications, possible management practices, and therapeutic outcomes of KD among the Iranian pediatric population.

## Methods

### Study design and participants

A cross-sectional retrospective study was designed and implemented with participating 190 patients with KD who were admitted to Bahrami Children Hospital (BCH; Tehran, Iran), affiliated with Tehran University of Medical Sciences (TUMS). The research was performed using census data from June 2008 to July 2018. The verbal and written informed consent was obtained from all the parents using phone contact and face-to-face interview methods after explaining the study objectives and used methodology. A single code number was assigned to each child to maintain the confidentiality of participants’ personal and medical information. The study framework entirely was approved by the Human Ethics Committee of the TUMS.

### KD diagnosis procedure

The diagnosis of complete and incomplete KD was based on standardized assessments such as medical history, physical examination, and laboratory measurements. In general, the KD diagnosis was made according to the diagnostic clinical criteria presented by the American Heart Association (AHA) [25, 26]. Patients with “complete” KD had four or more criteria, while patients with “incomplete” KD possessed two or three criteria [12]. The diagnosis in all patients with KD was made by a pediatric rheumatologist or a pediatric infectious disease specialist.

### Inclusion and exclusion criteria

All pediatric patients with the clinical diagnosis of KD who were admitted to BCH were included. On the other hand, KD children with the missing data in dataset were excluded from the study.

### Data collection

Demographic information including age, gender, the onset season of KD, mother’s pregnancy age, and childbirth method type (i.e., cesarean section or vaginal) was obtained from patients’ files.

The temperature degree and duration, other clinical manifestations and complications before and during hospitalization, and the first line of drug treatments were obtained from archived electronic files. Any type of infectious disease in a month before the appearance of symptoms was also considered as a positive history of recent infectious disease. The first line of treatment was defined as the first specific treatment for KD during admission and the second line of treatment included specific treatment for KD if the first line of treatment failed.

Laboratory data such as the count of white blood cells (WBCs), hemoglobin (Hb), and platelet (PLT), erythrocyte sedimentation rate (ESR), C-reactive protein (CRP), and levels of serum sodium at admission time were extracted from medical records of the hospital. Cardiac complications for KD were defined as the presence of CAAs, Kawasaki myocarditis, and valvular dysfunction characterized by echocardiography during follow-up visits. A pediatric cardiologist was performed the echocardiography in patients with KD. The involvement of the central nervous system (CNS) was evaluated based on the existence of restlessness, seizures, and sensorineural deafness. The hepatic complication was characterized by the presence of elevated liver enzymes of aspartate aminotransferase (AST) and alanine aminotransferase (ALT) (three times more than the upper normal range). Kidney involvement was diagnosed with increased creatinine, oliguria, or anuria. Gastrointestinal complications were characterized by nausea, vomiting, the presence of blood in the stool, and gallbladder hydrops. Also, joint complications were characterized by the presence of arthralgia or arthritis. Although the bacterial and viral etiology was not studied, 72.0 and 28.0% of the patients had a history of gastrointestinal disorders and upper respiratory tract infection (URTI), respectively. Macrophage activation syndrome (MAS) in Iranian children with KD was diagnosed according to criteria suggested by Ravelli et al. [27] with a combination of characteristic clinical features (such as non-remitting fever, generalized lymphadenopathy, hepatosplenomegaly, CNS dysfunction, and hemorrhagic manifestations) and typical laboratory abnormalities (such as pancytopenia, increased levels of ferritin, liver enzymes like lactate dehydrogenase, triglycerides, etc.) [21]. In this study, not only the mortality rate of children with KD was determined, but also the standardized mortality ratio (STM) was calculated. The STM was estimated according to the observed number of deaths divided by the expected number of deaths based on the vital statistics in Iran. Besides, 2.0 g/kg IVIG as an intravenous infusion was used during the first- and second-line treatment. Methylprednisolone was intravenously injected for three days at a dose of 30 mg/kg. A low-dose of ASA (3–5 mg/kg) was also used to treat patients with KD in the first-line treatment.

### Data analysis

The descriptive demographic data were represented as frequency, percentage, and mean ± standard deviation. Chi-square (χ^2^) was also carried out to examine the relationship between categorical qualitative variables (e.g., disease type (complete or incomplete) and complication type). The Kolmogorov–Smirnov test was used to assess the sample distribution normality. The Mann-Whitney U test was then used based on the non-normality of the quantitative data. The correlation between quantitative variables was assessed by the Spearman correlation coefficient given the absence of normal distribution of values. Moreover, possible clinical symptoms and laboratory findings to estimate the incidence of complications and the response to treatment were investigated using binomial and multinomial logistic models. The SPSS software package version 21.0 (SPSS Inc., Chicago, IL, USA) was used to analyze the data at a significant level of *p* < 0.05.

## Results

**Fig. 1:**
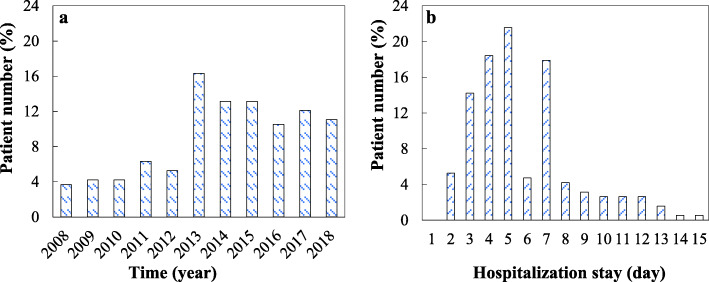
The percentage of KD patients in each year **a** and their hospitalization stay **b**

**Fig. 2:**
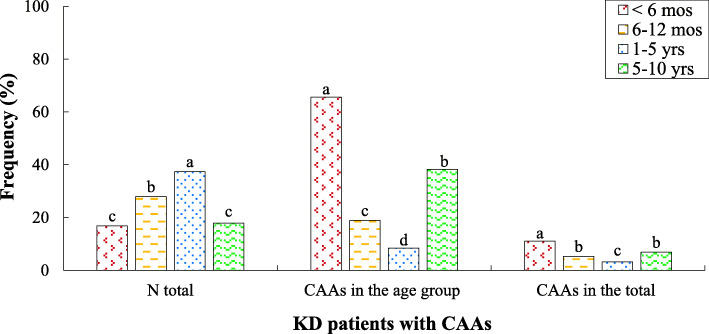
The KD patients diagnosed with CAAs in the total population and each age group. The means with dissimilar statistical letters **a**- are significantly different (*p* < 0.05)

23.68% of patients were hospitalized in the first five years of the study. After that, the number of patients significantly increased so that the average percentage of patients was 12.72% between 2013 and 2018 (Fig. 1a and b). 65.27% of the population had a complete form of KD, while the incomplete KD was diagnosed in the remaining cases (Table 1). Among 190 children with KD, 103 (54.21%) patients were boys and the remaining (45.79%) were girls, with a male-to-female ratio of 1.18:1. The mean age of the total population was 36 months, whereas the lowest and highest ages of patients were 1 month and 10 years, respectively. Patients according to the age were divided into four groups including < 6 months (*n* = 32, 16.84%), 6–12 months (*n* = 53, 27.90%), 1–5 years (*n* = 71, 37.37%), and 5–10 years (*n* = 34, 17.89%) (Fig. 2). Patients with complete and incomplete KD were not different in age (*p* = 0.054) and gender (*p* = 0.58). Also, no significant difference in the age of KD onset between boys and girls was found (*p* = 0.9). 63.7% of the patients (*n* = 121) were born with vaginal delivery. The mean gestational age at the delivery time of patients was 38.7 (±2.9) years. The frequency of patients in the season of KD onset over 10 years included spring (24.7%), summer (22.1%), autumn (26.8%), and winter (26.3%) (Table 1). Thus, no significant difference in was observed. 68.4% of the patients (*n* = 130) had a history of recent infection. The mean hospitalization duration of patients with KD was 5.4 (±2.2) days.

**Table 1:** Some demographic data and medical history of 190 Iranian children with KD

Demographic information	Freq. (%)
**Gender**	
Boy	54.21
Girl	45.79
**Mean age (month)**	36.00
**Season of KD onset**	
Spring	24.70
Summer	22.10
Autumn	26.80
Winter	26.30
**Medical history information**	
KD type - Complete	65.27
KD type - Incomplete	34.73
**Recent infection**	
Yes	68.42
No	31.58

Table 2 shows the frequency of clinical manifestations and complications in Iranian patients with KD. 92.6% of the patients were reported to have a fever. The average degree and duration of fever of the total population were 38.57 °C and 8.33 days, respectively. The percentage of patients with oral mucus membrane changes was 75.8%, including injected or fissured lips (32.6%), injected pharynx (20.5%), strawberry tongue (5.8%), and more than one type of involvement (16.9%). Furthermore, the percentage of bilateral bulbar conjunctival injection, polymorphous skin rash, peripheral extremity changes, and cervical lymphadenopathy was 73.7, 73.2, 63.7, and 60.0%, respectively (Table 2). Of the 190 Iranian children with KD, only 5 (2.63%; 3 boys, 2 girls) were diagnosed with MAS. In this study, the long-term mortality rate and STM of Iranian children with KD were determined to be 3 (two boys and 1 girl, 1.53%) and 0.51, respectively.

**Table 2:** The frequency of clinical manifestations and complications in Iranian patients with KD and their comparison with some data available in the literature

Manifestation/Complication type	Freq. (%)	Data obtained from the literature ^b^				
		Jindal et al. [17] India	Platt et al. [28] USA	Guleria et al. [29] India	Özdemir et al. [30] Turkey	Shamsizadeh et al. [24] Southwest of Iran
**Fever**	92.6	100	–	100	100	100
**Oral mucus membrane changes (Total)**	75.8	78.3	82.3	80.0	87.5	86.5
Injected or fissured lips	32.6	–	–	–	–	–
Injected pharynx	20.5	–	–	–	–	–
Strawberry tongue	5.8	–	–	–	–	–
More than one type	16.9	–	–	–	–	–
Bilateral bulbar conjunctival injection	73.7	63.0	85.8	57.1	87.5	89.4
Polymorphus skin rash	73.2	73.9	91.5	65.7	66.7	76.0
**Peripheral extremity changes (Total)**	63.7	–	–	–	50.0	66.3
Palms and soles erythema	41.6	–	–	–	–	–
Hands and feet edema	14.2	23.9	63.1	67.8	–	–
Periungual desquamation	1.1	97.8	–	91.4	20.8	–
More than one type	6.7		–	–	–	–
Cervical lymphadenopathy	60.0	60.9	39.6	60.0	70.8	42.3
**Gastrointestinal complications (Total)**	47.9	–	–	–	–	40.4
Diarrhea, nausea and vomiting	38.4	–	–	–	–	–
Blood in stool	8.0	–	–	–	–	–
Gastrointestinal bleeding requiring intervention	1.0	–	–	–	–	–
Gall bladder hydrops	0.5	–	–	–	12.5	–
**Cardiac complications (Total)**	27.9	–	–	–	–	–
Coronary artery aneurysm	26.3		–	–	–	–
Kawasaki myocarditis	1.6	6.5	–	–	–	–
Valvular involvement	0.0	–	–	–	–	–
**Joint involvement (Total)**	6.8	–	–	–	–	–
Arthralgia	4.8	–	–	–	–	14.4
Arthritis	2.0	19.6	–	80.0^a^	–	–
**Hepatic complication**	4.2	–	–	–	33.3	6.7

^a^ As oligoarticular involvement

Table 2 illustrates the frequency of complications encountered during the patients’ disease courses. 47.9% of the patients with KD had gastrointestinal complications, mainly diarrhea, nausea, and vomiting. Among these patients, two cases (1.0%) had gallbladder hydrops, while one patient (0.5%) with gastrointestinal bleeding required the emergent laparotomy and therapeutic intervention. Cardiac complications including CAAs (26.3%) and Kawasaki myocarditis (1.6%) were detected in 27.9% of the population. However, there was no significant correlation between patients’ age and the risk of coronary artery complications. Valvular involvement was observed in none of the patients. It was predictable that CAAs were the major cardiac complications among patients with KD. Figure 2 shows that the incidence of CAAs significantly was more in the age group below six months (*n* = 21 out of 32, 65.62%) compared to other age groups. The CAAs occurred in 10 (18.86%), 6 (8.45%), and 13 (38.23%) patients in age groups of 6–12 months, 1–5 years, and 5–10 years, respectively. In the total population, the lowest and highest KD children with CAAs were in age groups of below six months (11.05%) and 1–5 years (3.15%), respectively (*p* < 0.05, Fig. 2). Also, joint involvement (i.e., arthralgia and arthritis) and hepatic complication in the studied patients were determined to be 6.8% (*n* = 13) and 4.2% (*n* = 8), respectively. Thus, gastrointestinal and cardiac complications were the most common ones, while liver and joint complications less happened among Iranian pediatric patients with KD. In contrast, there was no significant difference in the age of patients with cardiac, gastrointestinal, and hepatic complications (Table 2). In this assessment, renal involvement or CNS involvement occurred in none of KD patients. The clinical manifestations of hands and feet edema and periungual desquamation in the present study occurred lower than other studies listed in Table 2. Boys were more likely to have gastrointestinal involvement in the course of the disease (*p* = 0.029). However, no significant differences were observed between different genders and the incidence of cardiac (*p* = 0.087) and hepatic (*p* = 0.228) involvements.

The analysis of haematological data shows that the mean amount of WBCs, Hb, PLT, ESR, CRP, and serum sodium was 11,567 N/μL, 10.6 g/dL, 527,618 N/mcL, 72.3 mm/h, 49.47 mg/L, and 138.5 mg/dL, respectively (Table 3). No significant difference in WBCs (*p* = 0.73), Hb (*p* = 0.53), ESR (*p* = 0.36), and CRP (*p* = 0.10) between patients with and without CAAs was found. However, KD cases with CAAs significantly had higher PLT counts (*p* = 0.01) and lower serum sodium levels (*p* = 0.002). A significantly higher CRP level in KD patients with hepatic involvement was detected (*p* < 0.001). But, no notable difference between KD patients with and without hepatic involvement were observed in the measured levels of WBCs (*p* = 0.83), PLT (*p* = 0.88), Hb (*p* = 0.64), ESR (*p* = 0.39), and serum sodium (*p* = 0.09). A similar result was also obtained for WBCs (*p* = 0.69), PLT (*p* = 0.14), Hb (*p* = 0.18), ESR (*p* = 0.35), CRP (*p* = 0.44), and serum sodium (*p* = 0.90) levels in KD patients with or without gastrointestinal involvement. Patients with complete KD compared to those with incomplete KD significantly had higher serum sodium levels (*p* = 0.03) and lower WBC counts (*p* = 0.02). Nonetheless, no significant difference was found between two groups in ESR (*p* = 0.63), CRP (*p* = 0.13), Hb (*p* = 0.15), and PLT (*p* = 0.10) amounts. Besides, the two groups were not different in cardiac (p = 0.8), gastrointestinal (*p* = 0.5) and hepatic (*p* = 0.14) complications.

**Table 3:** The laboratory markers data of Iranian children with KD compared to some data available in the literature

Marker type ^a^	Values (the present study)	Data obtained from the literature ^b^			
		Singh et al. [15]	Jindal et al. [17]	Platt et al. [28]	Özdemir et al. [30]
WBCs count (N/μL)	11,567 ± 6450	18.8 × 10^3^	13.45 × 10^3^	14.5 × 10^3^	16.3 × 10^3^
PLT count (N/mcL)	527,618 ± 238,427	710 × 10^3^	333 × 10^3^	355 × 10^3^	452 × 10^3^
Hb count (g/dL)	10.60 ± 1.31	nr	10.67	11.0	10.5
ESR (mm/h)	72.3 ± 30.0	37.9	42.5	65.0	77.0
CRP (mg/L)	49.7 ± 29.3	116.2	43.0	12.5	120.1
Sodium (mg/dL)	138.5 ± 3.5	nr	nr	nr	nr

^a^ *WBCs* white blood cells, *PLT* platelet, *Hb* hemoglobin, *ESR* erythrocyte sedimentation rate, *CRP* c-reactive protein

^b^ *nr* not reported

**Fig. 3:**
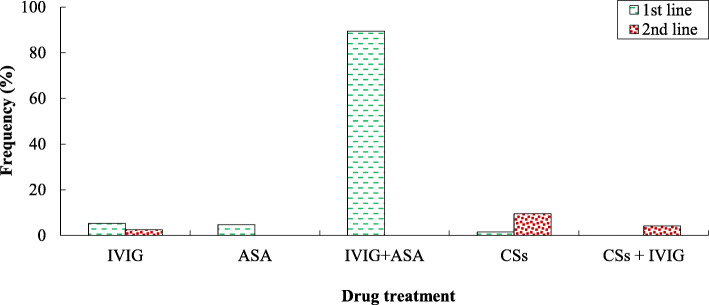
The used drugs in the first- and second-line treatments for Iranian children with KD

The evaluation of drug treatments shows that KD patients in the first line of treatment received IVIG + ASA (89.5%), IVIG (5.3%), ASA (4.7%), and CSs (0.5%). Besides, 16.3% of patients needed a second-line of treatment by receiving CSs (9.5%), CSs + IVIG (4.2%), and IVIG (2.6%) (Fig. 3). Patients who received IVIG in their treatment were more likely to have gastrointestinal involvements (*p* < 0.001). However, there was no significant difference in the occurrence of cardiac (*p* = 0.20) and hepatic (*p* = 0.67) involvements between cases that received different first-line treatments. Cardiac involvements significantly were higher in patients who needed second-line treatment (*p* < 0.001), but hepatic (*p* = 0.49) and gastrointestinal (*p* = 0.76) involvements significantly were not different. Based on the analysis of Spearman’s correlation test (95% CI), there was a significant correlation between hospitalization stay and Hb level (r = − 0.204, *p* = 0.005). Nevertheless, no significant relationship was found between other clinical and laboratory findings and the number of hospitalization days. The binomial logistic regression analysis was performed to determine a correlation between the need for second-line treatment and clinic-laboratory markers. There was a strong direct association between PLT count and need for second-line treatment (*p* = 0.003, r = 0.801). Also, a significant correlation was found between need for second-line treatment and levels of blood sodium (*p* = 0.007, r = − 0.838), and CRP (*p* = 0.023, r = 0.873).

## Discussion

KD is an acute systemic vasculitis affecting young children which its incidence is tremendously increasing worldwide. In this study, the number of boys with KD was more than girl patients. The gender difference in KD incidence was earlier observed in other studies conducted in the past decades in Iran [7, 23]. Similar results in other studies performed in Japan, Taiwan, the US, the United Kingdom (UK), Denmark, China, and Turkey were reported [31–35]. As the etiology of KD still is unknown, further studies are needed to investigate the cause of this gender predominance in KD. In this study, the mean age of KD patients was 36 months, while 83% of these individuals were under 5 y old. Similarly, 90, 72, 73, and 85% of KD patients in Taiwan, the UK, Denmark, and China were under the age of five, respectively [31, 33, 35]. A comparative assessment shows that the average age of patients in the US population was 36 months, while it was lower in the Chinese population (18 months) [32, 35]. Our results showed that the KD in cold seasons had a higher incidence. In contrast, the highest and lowest KD incidence among Taiwanese children was in the summer and winter seasons, respectively [31]. The maximum KD among Chinese children happened in the summer and spring seasons [10], while it more occurred in winter in Denmark and the UK [32, 34]. This finding suggests an environmental trigger for this clinical syndrome. Since a high number of the studied patients had a history of infectious diseases such as gastrointestinal disorders and URTI, the observed seasonal peak in the incidence of KD showed that these infectious diseases in the population might trigger this syndrome in different seasons [36]. In this study, 130 admitted patients with KD had an infection in the past month. Various studies have suggested that a viral infection is usually associated with KD. For instance, Benseler et al. [37] found that 33% of Canadian patients had at least one confirmed infectious disease at the diagnosis time of KD. Turnier et al. [38] reported that 41.9% of American children with KD showed positive respiratory viral polymerase chain reaction (PCR). They realized that rhinoviruses/enteroviruses were the most commonly detected viruses (28.1%) in these patients [38]. The presence of viral protein debris and inclusion of cytoplasmic cells containing viral sections by immunohistochemistry on the bronchial mucosa of KD patients were demonstrated by Rowley et al. [39]. There is still no study to determine the etiological relationship between viral diseases and KD. Epidemiologists have suggested there is a close association between the simultaneous occurrence of infectious diseases and KD. Therefore, further studies are needed to determine the viral etiology of KD.

The mean hospitalization stay of patients with KD in our study was 5.4 days. This indicator showed a significant, negative association with the Hb count so that patients with lower Hb number at admission time had more hospitalization days. This fact may be attributed to the prevalence of iron deficiency anemia in Iran and the lack of fortified foods, especially flour-based foods [40, 41]. An increase in hospitalization stay and major complications may slow down the healing process [41]. Also, the consumption of milk powders in feeding Iranian infants and lower-age children has been recently become common to replace breast milk. The lack of lactoferrin in traditional milk powders may lead to a reduction in Hb and an increase in the severity of disorders/complications, resulting in longer hospitalization [42, 43]. However, the Hb count in our study (10.6 g/dL) was in the range of data reported by other researchers (Table 3). Fischer et al. [34] reported that the average number of hospitalization days was 12 days for Danish children with KD. Ghimire et al. [44] have recently found that the average length of hospital stay for patients admitted on the weekend (4.1 days) was longer than those who were admitted on a weekday (3.72 days). Also, KD children with CAAs due to longer monitoring and management during the hospital setting had a longer hospital stay than patients without CAAs [44]. In this study, fever was the most common manifestation of KD as 92.6% of patients had a fever at admission time. However, other patients (7.4%) appeared fever during hospitalization. Other clinical manifestations were oral mucus membrane changes (75.8%), bilateral bulbar conjunctival injection (73.7%), polymorphous skin rash (73.2%), peripheral extremity changes (63.7%), and cervical lymphadenopathy (60.0%), respectively. Fever in studies conducted in Iran (92–98%) [7, 23] and other countries (up to 99.3%) [28, 31, 45] was also known as the principal manifestation. Similar results on the occurrence of other key manifestations were earlier reported [7, 23, 31]. The lower fever degree in our patients may be the result of prescribing antipyretics before visiting the hospital. In our study, the most common complication of patients with KD during hospitalization was gastrointestinal complications. Cardiac, joint, and liver complications after gastrointestinal ones had more frequencies in the population, respectively. Nakamura et al. [46] showed that the most frequent complications in Japanese children with KD were liver (27%), cardiac (25%), gallbladder inflammation (1.9%), and joint (1.1%), respectively. Huang et al. [35] reported 24.3% of KD cases in Shanghai had cardiac complications, whereas this rate in boys (27.1%) was more than girls (22.4%). More cardiac complications (33.3%) in Turkish children with KD were reported [46]. Cardiac complications (especially CAAs) in 20% of Iranian children with KD were earlier observed [7, 23]. In the present study, KD infants under six months of age were at a high risk of developing CAAs. This finding was in line with the results reported by other researchers [14, 15]. Singh et al. [15] concluded that incomplete forms of KD among Indian children in the same age group lead to a delay in diagnosing CAAs. A high risk of CAAs in KD children older than 10 y was reported by Jindal et al. [17]. We also realized the high risk of CAAs in older children aged 5–10 years. MAS was diagnosed in a low number of children with KD. Our results were in agreement with the findings of Pilania et al. [47]. They have recently reported that 1.3% of Indian children with KD (12 of the 950 cases) were diagnosed with MAS. Based on the collected data from 1994 to 2019, Guleria et al. [29] reported that 4.6% of 864 children diagnosed with KD had arthritis. Sadeghi et al. [23] also reported a high rate of arthralgia or arthritis (40%) and gallbladder hydrops (20%) in comparison to the obtained results in our study. On the other hand, the mortality rate in KD patients of the present study was comparable with that of children with KD mentioned by Singh et al. [48]. They reported four deaths (2 boys, 2 girls) among KD patients hospitalized in a tertiary care center in North India during 1994–2015.

There was no significant association between the type of KD (complete or incomplete) and its complications, which was consistent with the results obtained by Özdemir et al. [30]. We realized that there was no significant relationship between patients’ age and different complications (i.e., cardiac, gastrointestinal, joint, and liver). However, some researchers concluded that lower age significantly increased the heart damage risk among KD patients [14, 49]. Boys in our study more had gastrointestinal involvement in the course of the disease. There was no significant difference between the two genders in the incidence of cardiac and liver involvement. Nevertheless, Downie et al. [49] reported that gender was often associated with more occurrences of CAAs.

We did not find any significant difference in WBCs, Hb, ESR, and CRP between KD patients with and without CAAs. However, KD children with CAAs significantly had higher PLT counts and lower serum sodium levels. The number of PLT in our study was higher than the amounts reported by other researchers [17, 41, 50] (Table 3). Huang et al. [35] reported that CAAs significantly were correlated to lower ages of the KD onset, higher platelet counts, and lower serum albumin levels. The giant CAAs in patients with KD were earlier predicted with a serum sodium level of lower than 135 [50]. McCrindle et al. [51] also explained that the low serum levels of albumin and immunoglobulin M were associated with an increased risk of CAAs. CAAs-related risk factors in KD patients were assessed by Downie et al. [49] assessed. They included male gender, low age (under one year), and high PLT count.

89.5% of patients in the present study received drugs as first-line treatment for KD, while 16.3% of patients required second-line treatment. 70–100% of patients in other studies received IVIG as first-line treatment, whereas 12.5–16.5% of patients needed second-line treatment [7, 35, 46]. There was no significant correlation between the type of first-line treatment and cardiac complications in KD patients. Many studies have shown the effectiveness of IVIG treatment in reducing the incidence of cardiac complications. Some of the main mechanisms of IVIG in treating patients with KD include general immunosuppression through the generation modulation of pro-inflammatory cytokines, regulation of expression and function of Fc receptors, inhibition of complement activation, neutralization of bacterial superantigens or other infectious agents, and impacts on the activation, differentiation, and effector functions of T- and B cells, as well as other antigen-presenting cells like inhibition prevention of tumor necrosis factor-α production [17]. Also, the combination of IVIG with CSs or anti-inflammatory therapy such as ASA was associated with reduced cardiac complications [51–53]. More CAAs in the present study were associated with failure to respond to first-line treatment and the need for second-line drug treatment. Uehara et al. [54] also found non-response to the initial dose of IVIG was associated with an increased risk of CAAs. Higher counts of PLT and lower serum sodium and CRP levels in this study were associated with an increase in non-response to the first line of treatment and the need for the second line of treatment. According to the logistic model constructed by Kobayashi et al. [55], the day of illness at initial treatment, age in months, percentage of neutrophils, platelet count, and serum levels of AST, CRP, and sodium play a key role in determining the response to IVIG. Shamsizadeh et al. [24] pointed out that factors such as the male gender, receiving IVIG before the fifth day of illness, and Kawasaki re-emergence were significantly associated with non-response to IVIG.

### Study limitation

The sample size is one of the limitations of the present study. Hence, implementing a multicenter study with larger sample size is recommended to determine the risk factors related to KD complications and resistance to treatment. Also, one of the limitations of our retrospective study was the missing data in files such as more details about type of infections and aneurysm. Furthermore, another limitation was no classification of CAAs into the groups of small, medium-sized, and large (giant) aneurysms. Subsequent studies as a prospective cohort study at admission time of patients to the ward and their more accurate and longer follow-up will present a better understanding of the clinical features, complications of the disease, and response to treatment in patients with KD.

## Conclusions

This 10-year cross-sectional retrospective study showed that the increasing incidence rate of various manifestations of KD among Iranian children was similar to other parts of the world. The AHA’s diagnostic criteria for KD were efficient in identifying complete and incomplete KD among the Iranian pediatric population. There was a similar distribution of KD patients’ age and gender to European countries and the US. Although cardiac involvement was the main one, appropriate treatment and follow-up of patients would effectively prevent more complications. There is a necessity in determining risk factors for cardiac involvement. This assessment would be helpful in the diagnosis of high-risk individuals and their therapeutic and preventive measures. Higher PLT counts and lower levels of serum sodium and CRP can well predict failure to first-line treatment. This fact might make clinical judgments about the potential need for more effective or complementary treatment. However, further studies are needed to investigate the possible causes of treatment failure. On the other hand, the recognition and classification of CAAs may be a beneficial tool for risk stratification in the long-term management of children patients with KD. Therefore, a long-term follow-up should be performed for children with KD to assess a prognosis when there are moderate and severe cardiac complications.

## Data Availability

The dataset generated and analyzed during the study is available from the corresponding author on reasonable request.

## Abbreviations

ASA: Acetylsalicylic acid
ALT: Alanine aminotransferase
AST: Aspartate aminotransferase
CAA: Coronary artery aneurysm
CNS: Central nervous system
CRP: C-reactive protein
CSs: Corticosteroids
ESR: Erythrocyte sedimentation rate
Hb: Hemoglobin
IVIG: Intravenous immune globulin
KD: Kawasaki disease
PLT: Platelet
STM: Standardized mortality ratio
URTI: Upper respiratory tract infection
WBCs: White blood cells
